# Spatial intensity distribution analysis quantifies the extent and regulation of homodimerization of the secretin receptor

**DOI:** 10.1042/BCJ20170184

**Published:** 2017-05-24

**Authors:** Richard J. Ward, John D. Pediani, Kaleeckal G. Harikumar, Laurence J. Miller, Graeme Milligan

**Affiliations:** 1 Centre for Translational Pharmacology, Institute of Molecular, Cell and Systems Biology, College of Medical, Veterinary and Life Sciences, University of Glasgow, Wolfson Link Building 253, Glasgow G12 8QQ, U.K.; 2 Department of Molecular Pharmacology and Experimental Therapeutics, Mayo Clinic, 13400 East Shea Blvd, Scottsdale, AZ 85259, U.S.A.

**Keywords:** G-protein-coupled receptors, quaternary structure, spatial intensity distribution analysis

## Abstract

Previous studies have indicated that the G-protein-coupled secretin receptor is present as a homodimer, organized through symmetrical contacts in transmembrane domain IV, and that receptor dimerization is critical for high-potency signalling by secretin. However, whether all of the receptor exists in the dimeric form or if this is regulated is unclear. We used measures of quantal brightness of the secretin receptor tagged with monomeric enhanced green fluorescent protein (mEGFP) and spatial intensity distribution analysis to assess this. Calibration using cells expressing plasma membrane-anchored forms of mEGFP initially allowed us to demonstrate that the epidermal growth factor receptor is predominantly monomeric in the absence of ligand and while wild-type receptor was rapidly converted into a dimeric form by ligand, a mutated form of this receptor remained monomeric. Equivalent studies showed that, at moderate expression levels, the secretin receptor exists as a mixture of monomeric and dimeric forms, with little evidence of higher-order complexity. However, sodium butyrate-induced up-regulation of the receptor resulted in a shift from monomeric towards oligomeric organization. In contrast, a form of the secretin receptor containing a pair of mutations on the lipid-facing side of transmembrane domain IV was almost entirely monomeric. Down-regulation of the secretin receptor-interacting G-protein Gα_s_ did not alter receptor organization, indicating that dimerization is defined specifically by direct protein–protein interactions between copies of the receptor polypeptide, while short-term treatment with secretin had no effect on organization of the wild-type receptor but increased the dimeric proportion of the mutated receptor variant.

## Introduction

The concept that single-polypeptide G-protein-coupled receptors (GPCRs) can form, exist and may function as dimers and/or higher-order oligomers, rather than simply as monomers, has steadily gained acceptance [1,2]. In the case of the glutamate-like or ‘class C’ receptors, their existence as obligate dimers/oligomers has long been established [3] and is an integral component of their mechanism of signal transduction. In contrast, for the rhodopsin-like or ‘class A’ GPCRs, clear evidence shows that they can function as monomers [4,5], and this has led to debate over the relevance of the function and pharmacology of dimers and/or higher-order oligomers, which clearly can be shown to exist [1,2,6], even if in some cases the stability and durability of such quaternary structures have been questioned [1,7,8] or even argued to be incorrect [9]. As with many other areas of study on the structure, function and pharmacology of the over-arching GPCR superfamily, questions relating to the organizational structure of the small group of ‘class B’ receptors, which recognize and respond to sets of peptide hormones that control many aspects of integrative physiology, have attracted rather less attention [10]. Despite this, many of the class B receptor members have been actively studied in relation to their quaternary structure, with perhaps the greatest attention to date having been directed towards the secretin receptor [11–14]. As for many class A GPCRs, the application of resonance energy transfer (RET)-based techniques, in which forms of a receptor labelled with distinct, RET-competent tags are co-expressed, has been central to analysis of ‘dimerization’ and for the secretin receptor, combinations of bioluminescence resonance energy transfer (BRET) and bi-molecular fluorescence complementation (Bi-FC) have been used to suggest that this receptor exists as dimers but not as higher-order oligomers [13]. Moreover, by employing a series of peptides corresponding to individual transmembrane domains of the secretin receptor to disrupt potential receptor–receptor interactions, Harikumar et al. [14] were able to provide evidence of a key role of transmembrane domain IV in forming the dimer interface. Moreover, mutation of residues of transmembrane domain IV predicted to be on the external, lipid-facing region of this sequence resulted in abrogation of receptor–receptor interactions measured by RET techniques [14]. The minimum set of mutations required to produce this effect was conversion of both residues Gly^243^ and Ile^247^ into Ala [14]. In addition to the effects upon dimerization, it was observed that these mutations also reduced the potency of secretin to stimulate intracellular cyclic AMP (cAMP) production [14].

RET-based techniques have provided enormous insights into potential protein–protein interactions in living cells and how these may be disrupted [15–17], but clear limitations apply for RET, not least the very specific effects of distance and dipole orientation on signals observed. Moreover, although widely used in transfected cell systems, the production of BRET- or fluorescence resonance energy transfer (FRET)-competent partners generally requires molecular manipulation to introduce fluorescent proteins and/or a bioluminescent enzyme into the sequence of the protein being studied, and as such would require complex genetic engineering and crossing of transgenic lines to generate animals in which such studies could be performed with receptors expressed at endogenous levels [15]. Moreover, as RET techniques require close proximity or direct interaction between copies of the differentially labelled variants of the protein of interest, while it is suitable to define that some proportion of the protein is dimeric or oligomeric, it is not well suited to assess what this proportion may be, and, as such, whether a significant fraction of the secretin receptor is monomeric remains undefined. In recent years, there have been efforts to employ mathematical analysis of the distribution of fluorescent signal generated from a single fluorophore-tagged protein to gain insights into the quaternary organization of such tagged proteins. Spatial intensity distribution analysis (SpIDA) has been suggested to be able to do so [18,19], and in recent times we have shown that SpIDA can indeed identify both monomeric versus dimeric/oligomeric organization, and ligand regulation of such organizational structure, simply by appropriate mathematical analysis of regions of interest (RoIs) within laser scanning confocal images from cells expressing a single protein construct tagged with a suitable fluorophore [20–22]. In the current studies, we have applied SpIDA to reassess the basal organizational structure of the secretin receptor at different expression levels and the effects of mutations within transmembrane domain IV on this, as well as to probe the contribution of G-protein to the stability of secretin receptor organization and the potential of the ligand secretin to alter this. To enhance confidence in the quantitative analyses, we also generated and tested a variety of other protein constructs, labelled with the same monomeric form of enhanced green fluorescent protein (mEGFP), that are known to exist as monomers or dimers or are known to interconvert between these states in a ligand-dependent manner [20–22]. The results indicate that, at moderate expression levels, the basal state of the secretin receptor is predominantly dimeric, although a significant proportion of monomers coexist with the dimers, and that increase in the expression level decreases the proportion of monomeric species while leading to the detection of oligomeric forms that are larger than dimers. Mutation of key residues in transmembrane domain IV alters this equilibrium to greatly favour the monomeric state. Extensive down-regulation of Gα_s_, the key signal transducing G-protein associated with secretin receptor function, had no effect on the distribution of receptor quaternary structure while, although without effect on the structure of the wild-type receptor, short-term addition of secretin increases the organizational structure of the mutationally modified form of the receptor that compromises basal dimerization.

## Experimental procedures

### Materials

General laboratory chemicals were from Sigma–Aldrich (Poole, U.K.) or Fisher Scientific (Leicester, U.K.). Otherwise, DNA restriction endonucleases, calf intestinal alkaline phosphatase, T4 DNA polymerase and T4 ligase were from New England Biolabs (Hitchin, U.K.). The Wizard Plus SV Miniprep kit was from Promega (Southampton, U.K.). NuPage Novex precast 4–12% Bis–Tris gels, NuPage MOPS SDS running buffer, NativePAGE™ Novex® 3–12% Bis–Tris Gels and associated reagents were from Invitrogen (Paisley, U.K.). QIAfilter Plasmid Maxi Kit, PCR purification kit and QIAquick gel extraction kit were from Qiagen (Crawley, U.K.). Agarose was from Flowgen Biosciences (Nottingham, U.K.). Secondary horseradish peroxidase-conjugated antibody was from Sigma–Aldrich (Poole, UK) or GE Healthcare Life Sciences (Buckinghamshire, UK). ECL reagent was purchased from Pierce (Tattenhall, U.K.). Polyethylenimine was from Polysciences, Inc. (Warrington, PA, U.S.A.). Protease inhibitor cocktail tablets and *N*-glycosidase F were from Roche Diagnostics, (Mannheim, Germany). Antibodies were either generated ‘in house’ (anti-GFP and anti-Gα_s_) or were from Santa Cruz Biotechnology [Insight Biotechnology, Middlesex, U.K.; anti-epidermal growth factor receptor (EGFR)], Sigma–Aldrich (Poole, UK) (anti-tubulin) or Abcam (Cambridge, U.K.) [anti-EGFR (phospho-Y1068), anti-Na^+^/K^+^ATPase]. Cholera toxin and *n*-dodecyl β-d-maltoside were from Sigma–Aldrich (Poole, U.K.). Secretin was from Tocris Bioscience (Bristol, U.K.).

### DNA constructs

PM-1-mEGFP, PM-2-mEGFP and EGFR-mEGFP were made as described in ref. [20]. A C-terminally truncated form of Robo 1-mEGFP was made as described in ref. [21]. Tyr^251^Ala,Arg^285^Ser EGFR-mEGFP [23] was a modification of the EGFR-mEGFP construct, made by two rounds of Dpn1 mutagenesis (QuikChange, Agilent Technologies/Stratagene, Santa Clara, U.S.A.). Human secretin receptor-mEGFP and human Gly^243^Ala,Ile^247^Ala secretin receptor-mEGFP constructs were made from constructs as described in ref. [14] by inserting fragments, cut with HindIII (then blunted, using T4 DNA polymerase) and Xho1, corresponding to secretin receptor and Gly^243^Ala,Ile^247^Ala secretin receptor into pEGFP-N1 (Takara Bio Europe/Clontech, Saint-Germain-en-Laye, France) at Nhe1 (blunted, as above) and Xho1. All constructs containing mEGFP incorporated the Ala^206^Lys mutation to ensure that the results were not influenced by any tendency of the fluorescent protein to aggregate [24]. All constructs were verified by sequencing.

### Cell lines

All cells were maintained in a humidified incubator with 95% air and 5% CO_2_ at 37°C. Parental Flp-In™ T-REx™ 293 cells (Invitrogen, Paisley, U.K.) were maintained in DMEM (high glucose) supplemented with 10% (v/v) foetal bovine serum, 100 U ml^−1^ penicillin, 100 µg ml^−1^ streptomycin, 10 µg ml^−1^ blasticidin and 100 µg ml^−1^ zeocin. Cell lines generated that used Flp-In™ T-REx™ 293 cells as the base were maintained in DMEM (high glucose) supplemented with 10% (v/v) foetal bovine serum, 100 U ml^−1^ penicillin, 0.1 mg ml^−1^ streptomycin, 10 µg ml^−1^ blasticidin and 200 µg ml^−1^ hygromycin B. Chinese hamster ovary (CHO) secretin receptor-mEGFP and CHO Gly^243^Ala,Ile^247^Ala secretin receptor-mEGFP cell lines were maintained in Hams F-12 Nutrient Mix (Invitrogen, Paisley, U.K.) supplemented with 5% (v/v) foetal bovine serum, 100 U ml^−1^ penicillin, 100 µg ml^−1^ streptomycin and 500 µg ml^−1^ zeocin.

### Stable cell line generation

Inducible Flp-In™ T-REx™ stable cell lines capable of expressing PM-1-mEGFP, PM-2-mEGFP, EGFR-mEGFP, Tyr^251^Ala,Arg^285^Ser EGFR-mEGFP and the Robo 1-mEGFP construct were generated as described [20–22]. Briefly, 48 hrs after transfection, the medium was changed to that with no zeocin, but supplemented with 200 µg ml^−1^ hygromycin to initiate the selection of stably transfected cells. Pools of cells were established (10–14 days for resistant colonies to form) and tested for inducible expression by the addition of 100 ng ml^−1^ doxycycline for 48 h followed by screening for fluorescence corresponding to mEGFP and by immunoblotting.

mEGFP-tagged wild-type and Gly^243^Ala,Ile^247^Ala secretin receptor-bearing CHO cell lines were prepared by transfection of CHO cells known to not express any secretin receptors, following the approach we previously described [25]. Stable, receptor-bearing cells were enriched using zeocin (500 µg ml^−1^) and clonal cell lines were selected utilizing limiting dilution techniques. These were shown to exhibit fluorescence at the cell surface and levels of receptor expression were quantified using secretin radioligand binding and by quantification of mEGFP fluorescence.

### Cell membrane preparation

Cells induced with the required concentration of doxycycline to express PM-1-mEGFP, PM-2-mEGFP, Robo1(truncated)-mEGFP or other constitutively expressing constructs were washed and then harvested with ice-cold phosphate-buffered saline (PBS). Pellets of cells were frozen at −80°C for a minimum of 1 h, thawed and resuspended in ice-cold 10 mM Tris, 0.1 mM EDTA (pH 7.4, TE buffer) supplemented with the Complete protease inhibitor cocktail. Cells were homogenized on ice by 40 strokes of a glass-on-Teflon homogenizer followed by centrifugation at 1000×***g*** for 5 min at 4°C to remove unbroken cells and nuclei. The supernatant fraction was removed and passed through a 25-gauge needle 10 times before being transferred to ultracentrifuge tubes and subjected to centrifugation at 50 000×***g*** for 30 min at 4°C. The resulting pellets were resuspended in ice-cold TE buffer, the protein concentration was assessed and membranes were stored at −80°C until required.

### Generation of cell lysates and immunoblotting

Cells were washed once in cold PBS [120 mM NaCl, 25 mM KCl, 10 mM Na_2_HPO_4_ and 3 mM KH_2_PO_4_ (pH 7.4)] and harvested with a minimum volume ice-cold lysis buffer [150 mM NaCl, 0.01 mM Na_3_PO_4_, 2 mM EDTA, 0.5% *n*-dodecyl β-d-maltoside and 5% glycerol plus Protease inhibitor cocktail tablets (pH 7.4)]. Extracts were passed through a 25-gauge needle and incubated for 30 min at 4°C while on a rotating wheel, centrifuged for 10 min at 21 000×***g*** and the supernatant was recovered to fresh tubes. Samples were prepared by the addition of SDS–PAGE sample buffer and heated to 65°C for 5 min before being subjected to SDS–PAGE analysis using NuPAGE 4–12% Bis–Tris gels and MOPS buffer. After separation, the proteins were electrophoretically transferred to the nitrocellulose membrane, which was then blocked (5% fat-free milk powder in PBS with 0.1% Tween-20) at 4°C on a rotating shaker overnight. The membrane was incubated for 3 h with primary antibody [1 : 10 000 sheep anti-GFP, 1 : 10 000 rabbit anti-Gαs, 1 : 1000 rabbit anti-EGFR, 1 : 10 000 rabbit monoclonal anti-EGFR (phospho-Y1068), as indicated] in 2% fat-free milk powder in PBS-Tween, washed (3 × 10 min PBS-Tween) and then incubated for 3 h with appropriate secondary antibody (horseradish peroxidase-linked rabbit anti-goat IgG or donkey anti-rabbit, diluted 1 : 10 000 in 2% fat-free milk powder in PBS-Tween). After washing as above, the signal was detected by enhanced chemiluminescence (Pierce Chemical, Rockford, IL) according to the manufacturer's instructions. Alternatively, some blots were detected using fluorescently labelled secondary antibodies [IRDye® 800CW donkey anti-goat and IRDye® 680RD donkey anti-mouse (Li-Cor Biotechnology, Cambridge, U.K.). These were used at a dilution of 1 : 20 000 in Odyssey® PBS blocking buffer (Li-Cor Biotechnology) according to the manufacturer's instructions. Secondary antibodies were detected using an Odyssey Sa Infra-red Imaging System (Li-Cor Biotechnology).

### Native blue polyacrylamide gel electrophoresis

Flp-In™ T-REx™ 293 cells, induced with doxycycline to express constructs as indicated and subjected to treatments as indicated, were harvested in 1× PBS and lysed in lysis buffer [150 mM NaCl, 0.01 mM Na_3_PO_4_, 2 mM EDTA, 0.5% *n*-dodecyl β-d-maltoside and 5% glycerol supplemented with Protease inhibitor cocktail tablets (pH 7.4)] on a rotating wheel for 30 min at 4°C. Samples were then centrifuged for 30 min at 100 000×***g*** at 4°C and the supernatants were collected. Solubilized supernatant (16 µg) plus 4 µl of G250 additive was loaded on to each lane of NativePAGE™ Novex® 3–12% Bis–Tris Gels. In some samples, SDS was added to 1% final concentration 10 min prior to loading. After electrophoresis at 0°C (using buffers and conditions indicated by the manufacturer), proteins were transferred (90 min at 25 V) onto a PVDF membrane, which had been pre-wetted for 30 s in methanol and then soaked for several minutes in transfer buffer. The membrane was then fixed in 8% acetic acid, shaken for 15 min, stained with Ponceaux S (Sigma–Aldrich, Poole, U.K.; 0.2% in 1% acetic acid) to allow the markers to be visualized, rinsed to remove the Ponceaux S and immunoblotted with anti-GFP antiserum as described above.

### cAMP assays

cAMP assays were performed using a homogeneous time-resolved fluorescence (HTRF®) cAMP dynamic kit (CisBio Bioassays, Codolet, France). Cells were detached by incubation at 37°C for 5–10 min with Versene (Invitrogen, Paisley, U.K.), counted and added at 5000 cells/well to low-volume 384-well plates (Proxi-plate™ 384 Plus, PerkinElmer Life Sciences). cAMP production was stimulated by the addition of varying concentrations of secretin followed by a 30 min incubation at room temperature. Outputs were measured by using a PHERAstar FS plate reader (BMG Labtech Ltd, Buckinghamshire, U.K.).

### Determination of relative expression levels by mEGFP fluorescence

CHO cell lines expressing secretin receptor-mEGFP or Gly^243^Ala,Ile^247^Ala secretin receptor-mEGFP were seeded at 25 000 cells per well in 96-well solid black bottom plates (Greiner Bio-One) pre-coated with 0.1 mg ml^−1^ poly-d-lysine. After 24 h growth, cells were washed three times in HBSS buffer (Invitrogen, Paisley, U.K.). A 100 µl aliquot of HBSS was added to each well and the plates were read using a CLARIOstar fluorescence-compatible reader (BMG Labtech Ltd, Buckinghamshire, U.K.) using an excitation wavelength of 488 nm and an emission wavelength of 535 nm. The HBSS was then replaced with a further 100 µl/well of HBSS containing a 1 : 1000 dilution of 10 mg ml^−1^ Hoechst 33342 stain (Molecular Probes, OR, U.S.A.). The plate was incubated at 37°C for 15 min, and the HBSS and stain were then removed and the wells washed with 2 × 100 µl HBSS; 100 µl/well of HBSS was added and the plate was read using an excitation wavelength of 355 nm and an emission wavelength of 455 nm. The second reading was then used to correct the first for any variations in cell number.

### Deglycosylation reactions

Cells were washed once and harvested in cold PBS. Pellets were incubated in lysis buffer [150 mM NaCl, 0.01 mM Na_3_PO_4_, 2 mM EDTA (pH 7.4), 0.5% *n*-dodecyl β-d-maltoside and 5% glycerol, supplemented with Complete protease inhibitor cocktail tablets, Roche Diagnostics), on a rotating wheel for 1 h at 4°C. Samples were then centrifuged at 4°C for 15 min at 14 000×***g***, and the supernatant was aliquoted and its protein concentration was assessed, before storage at −80°C. Deglycosylation reactions were set up by adding 1 unit of *N*-glycosidase F to 15 µg of protein lysate and 1× *N*-glycosidase F buffer (20 mM phosphate buffer, pH 7.2) in a final volume of 15 µl. This was then incubated for 2 h at 37°C before being analyzed by SDS–PAGE.

### Spatial intensity distribution analysis

SpIDA was carried out essentially as described in ref. [20]. All RoIs measurements were selected from the basolateral membrane surface. Monomeric equivalent unit (MEU) values for EGFR-mEGFP, Tyr^251^Ala,Arg^285^Ser EGFR-mEGFP, Robo1-mEGFP, secretin receptor-mEGFP and Gly^243^Ala,Ile^247^Ala secretin receptor-mEGFP were measured by normalizing their assessed quantal brightness (QB) values with an average QB value measured from the PM-1-mEGFP construct (all measurements were made using 2% laser power to ensure consistency). To distinguish between monomeric and dimeric/oligomeric species, PM-1-mEGFP MEU occurrence/frequency *x*–*y* graphs (MEU bin size = 0.2) were plotted for each MEU value measured during excitation. Such plots revealed a symmetrical distribution of the values, and Graphpad Prism normality tests indicated that the distributions were Gaussian (see Results and Statistical analyses). The data from each frequency *x*–*y* plot of EGFR-mEGFP, Tyr^251^Ala,Arg^285^Ser EGFR-mEGFP, Robo1-mEGFP, secretin receptor-mEGFP and Gly^243^Ala,Ile^247^Ala secretin receptor-mEGFP were then divided at an MEU value of 1.48 (which represented 75% of the data set, falling within the mean + 1.5 SD), which was set as the border to distinguish between monomeric and dimeric species in studies where individual MEU values exceeded 1.48.

In the case of EGFR-mEGFP and Tyr^251^Ala,Arg^285^Ser EGFR mEGFP cells were grown in Lab-Tek 4-well-chambered cover glasses (Thermo Fisher Scientific, Paisley, U.K.), treated (or not) with EGF for 10 min and then fixed with 4% paraformaldehyde. The cells were kept under PBS at 4°C prior to imaging. Similarly, secretin receptor-mEGFP and Gly^243^Ala,Ile^247^Ala secretin receptor-mEGFP cells were also grown in 4-well-chambered coverslips prior to treatment (or not) with secretin (concentration and times as indicated) before fixation and storage as above.

### Calculation of receptor density at the cell surface by SpIDA

The SpIDA software also reports the mean fluorescence intensity for each RoI analyzed. The number of EGFR-mEGFP, Tyr^251^Ala,Arg^285^Ser EGFR-mEGFP, Robo1-mEGFP, secretin receptor-mEGFP and Gly^243^Ala,Ile^247^Ala secretin receptor-mEGFP molecules µm^−2^ (density) was measured by dividing the mean fluorescence intensity value by the quantified monomeric QB value.

### Statistical analyses

Variation in receptor number or mean/median of QB produced by treatment with either ligands or with varying concentrations of doxycycline was assessed by either Student's *t*-test or one-way ANOVA, with the use of Bonferroni's or Dunnett's test for multiple comparisons as appropriate. Normality distributions of recovered QB values defined as MEUs were assessed by D'Agostino and Pearson, normality tests (at *P* > 0.05) and by skewness and Kurtosis assessments. Distributions that failed the normality assessment (at *P* < 0.05) were considered to be non-Gaussian.

## Results

**Figure 1.**
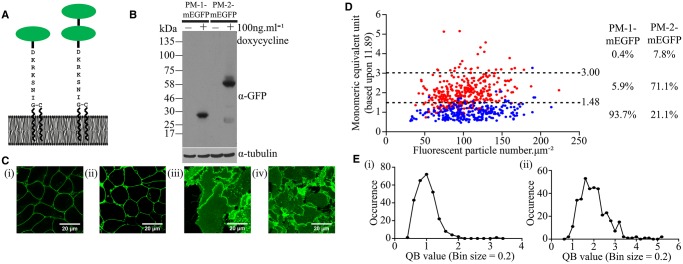
Calibration of SpIDA analysis using plasma membrane-attached forms of monomeric EGFP. Cartoon illustration of the plasma membrane interaction of PM-1-mEGFP and PM-2-mEGFP constructs. The green ellipses signify molecules of mEGFP (**A**). (**B**) Immunoblot analysis of the membrane expression of PM-1-mEGFP and PM-2-mEGFP following doxycycline-induced expression in Flp-In™ T-REx™ 293 cells using an anti-GFP antiserum. Corresponding immunodetection of tubulin provided loading controls. (**C)** Confocal images of Flp-In™ T-REx™ 293 cells expressing either PM-1-mEGFP (**i,iii**) or PM-2-mEGFP (**ii,iv**) across the centre of cells (**i,ii**) or at the basolateral surface (**iii,iv**). Scale bar = 20 µm. (**D**) SpIDA analysis displaying the full data set captured from individual RoIs presented as MEUs versus levels per µm^2^. Blue: PM-1-mEGFP; red: PM-2-mEGFP. Values corresponding to mean + 1.5 SD are shown (1.48 MEU for PM-1-mEGFP and 3.00 MEU for PM-2-mEGFP) as dotted lines. Proportions of each construct scored as monomer, dimer and oligomer are shown. (**E**) Individual QB values, presented as MEU, for both PM-1-mEGFP (**Ei**) and PM-2-mEGFP (**Eii**) displayed Gaussian distribution.

SpIDA can sample confocal laser scanning images to discriminate between distributions of monomers and dimers/oligomers of proteins tagged with EGFP within defined RoIs [18–22]. We initially employed Flp-In™ T-REx™ 293 cells stably harbouring at the Flp-In™ T-REx™ locus either a single copy of monomeric Ala^206^Lys EGFP (mEGFP) [24] or a linked tandem of this polypeptide, each form containing an introduced, plasma membrane-anchoring, palmitoylation + myristoylation (P–M) sequence taken from the N-terminus of the Lyn non-receptor tyrosine kinase (Figure 1A). In each case, the addition of the antibiotic doxycycline allowed expression of the corresponding protein (Figure 1B) and imaging of the cells confirmed location of the polypeptides at the plasma membrane (Figure 1C(i, ii)). RoIs within confocal images of the coverslip-attached basal surface of such cells (Figure 1C(iii,iv)) were subjected to QB measurement and SpIDA (Figure 1D). By varying the concentration of the inducer doxycycline, a range, from 32–214 molecules µm^−2^ (mean 107.9 ± 2.1, *n* = 270) (Table 1), of amounts of the PM-1-mEGFP construct was observed (Figure 1D). Importantly, analysis of the PM-1-mEGFP construct across this range of expression levels indicated that the QB values (mean 11.89 ± 0.23 units, *n* = 270) were normally distributed (Figure 1E(i)); therefore, as a monomeric protein, this mean QB value was defined as corresponding to 1.00 MEU (Figure 1E(i)). The analysis of the full data set indicated that mean + 1.5 SD of the QB values for PM-1-mEGFP corresponded to 1.48 MEU. In subsequent studies, QB values >1.48 MEU were therefore defined as ‘larger than monomer’ (see Experimental for details). Equivalent studies were also performed on RoIs selected from confocal images of the basolateral surface of cells induced to express PM-2-mEGFP, i.e. the construct containing the tandemly linked pair of mEGFP molecules. This resulted in expression levels (38–224 molecules µm^−2^, mean 101.4 ± 1.5) that were equivalent to those of PM-1-mEGFP (Figure 1D). Now, however, the absolute QB (mean 23.74 ± 0.42 units, *n* = 360) was 1.99 ± 0.04 times the mean QB recorded for PM-1-mEGFP. This indicates that the tandemly linked pair of mEGFP molecules is identified in this approach, on average, as corresponding to a ‘dimer’. Analysis of the full data set showed that once more the QB values were normally distributed (Figure 1E(ii)). Here, mean + 1.5 SD of the QB corresponded to 3.00 MEU and, therefore, in subsequent studies, QB values >3.00 MEU were considered to be ‘larger than dimer’ (i.e. oligomeric) (Figure 1D). Using this analysis, for PM-1-mEGFP, 93.7% of the observations corresponded to ‘monomer’ and 5.9% to ‘dimer’, with <0.5% identified as ‘oligomer’. For PM-2-mEGFP, 71.1% of the observations corresponded to ‘dimer’, 7.8% to ‘oligomer’ and 21.1% to ‘monomer’ (Figure 1D).

**Table 1:** Details of analysis of mEGFP-tagged control constructs used

Construct and treatment	Number of RoIs analyzed	Mean MEU value	Range of expression (µm^−2^)	Average expression (µm^−2^)
PM-1-mEGFP	270	1.00 ± 0.02	32–214	107.9 ± 2.1
PM-2-mEGFP	360	1.99 ± 0.04	38–224	101.4 ± 1.5
EGF-R-mEGFP (UT)	240	1.22 ± 0.33	29–135	55.0 ± 1.2
EGF-R-mEGFP (100 nM EGF)	240	1.97 ± 0.03	20–121	62.1 ± 1.4
EGF-R Y^251^A R^285^S-mEGFP (UT)	240	1.14 ± 0.02	22–156	56.2 ± 1.3
EGF-R Y^251^A R^285^S-mEGFP (100 nM EGF)	240	1.11 ± 0.02	24–140	53.1 ± 1.3
(Truncated) Robo1-mEGFP	128	2.04 ± 0.05	20–128	48 ± 1.2

Abbreviation: UT, untreated.

**Figure 2.**
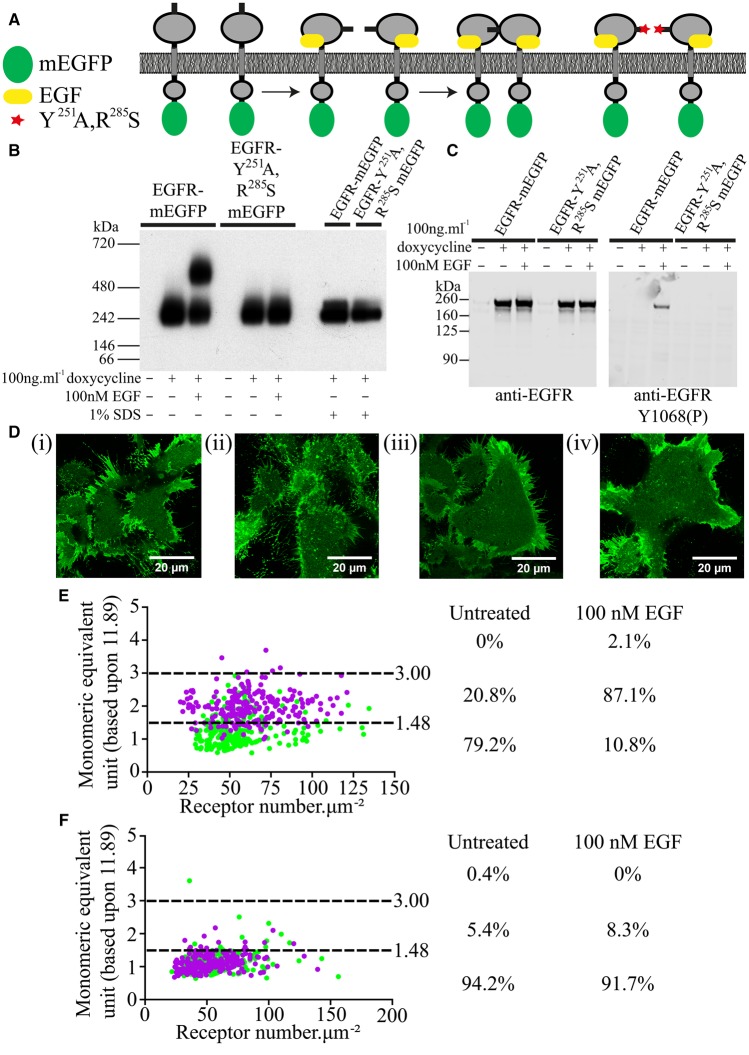
The EGFR undergoes ligand-induced dimerization, while Tyr^251^Ala, Arg^285^Ser EGFR does not. Cartoon illustrating the potential for the binding of EGF (yellow) to promote dimerization of wild-type EGFR, while the mutations (red) in Tyr^251^Ala,Arg^285^Ser EGFR prevent this (**A**). (**B**) Blue native PAGE analysis of wild-type EGFR and Tyr^251^Ala,Arg^285^Ser EGFR treated or not with EGF (100 nM, 10 min). In the two right-hand lanes, samples were treated with 1% (w/v) SDS prior to the addition to the gel. Lanes labelled — Dox were not induced to express the receptor constructs. Results show anti-GFP immunostaining. (**C**) EGF promotes phosphorylation of Tyr^1068^ in wild-type EGFR but not in the Tyr^251^Ala, Arg^285^Ser mutant. Samples resolved by SDS–PAGE and immunoblotted to detect either total EGFR levels (**left-hand side**) or Tyr^1068^ phosphorylation (**right-hand side**). (**D**) Basolateral images of Flp-In™ T-REx™ 293 cells induced to express wild-type EGFR (**i,ii**) or Tyr^251^Ala, Arg^285^Ser EGFR (**iii,iv**) treated with vehicle (**i,iii**) or EGF (**ii,iv**). (**E**) SpIDA of RoIs from cells expressing wild-type EGFR, untreated (**green**) or treated with EGF (**purple**). (**F**) SpIDA of RoIs from cells expressing Tyr^251^Ala, Arg^285^Ser EGFR, untreated (**green**) or treated with EGF (**purple**). Proportions of each construct scored as monomer, dimer and oligomer are shown.

To provide further confidence in the capacity of SpIDA to define monomeric, dimeric and oligomeric populations of a true transmembrane receptor protein, we turned to the wild-type EGFR and a Tyr^251^Ala,Arg^285^Ser mutant of this receptor [23]. The wild-type EGFR is known to move rapidly from being largely monomeric to being predominantly dimeric upon binding of the ligand EGF, while the Tyr^251^Ala,Arg^285^Ser mutant fails to dimerize or signal in response to the ligand (Figure 2A) [23]. To confirm this expectation, we generated Flp-In™ T-REx™ 293 cells stably harbouring either wild-type EGFR-mEGFP or Tyr^251^Ala,Arg^285^Ser EGFR-mEGFP. Following induction of expression with doxycycline, cells were treated with or without 100 nM EGF for 10 min, cell lysates were generated, resolved by non-denaturing Blue native PAGE and immunoblotted with an anti-GFP antiserum (Figure 2B). As shown recently [20], the addition of EGF to the wild-type receptor resulted in a substantial proportion of this polypeptide now migrating as a dimer, while in the absence of EGF all of the detected receptor was monomeric. However, although expressed at similar levels as the wild-type receptor, Tyr^251^Ala,Arg^285^Ser EGFR-mEGFP remained monomeric after the addition of EGF (Figure 2B). Importantly, the dimeric form of EGFR-mEGFP did not represent a covalently linked adduct. The addition of SDS to samples prior to resolution by Blue native PAGE resulted in all of both wild-type and Tyr^251^Ala,Arg^285^Ser EGFR-mEGFP migrating as monomeric species. The lack of activation of Tyr^251^Ala,Arg^285^Ser EGFR-mEGFP by EGF was also highlighted following resolution of untreated and EGF-treated samples by denaturing SDS–PAGE, where although the wild-type receptor became phosphorylated on residue Tyr^1068^ in response to the ligand, Tyr^251^Ala,Arg^285^Ser EGFR did not (Figure 2C). RoIs of confocal images from the basolateral surface of cells induced to express wild-type EGFR-mEGFP (Figure 2D) were analyzed via the SpIDA software. This showed that across expression levels of 29–135 molecules µm^−2^, while this construct was detected predominantly as a monomer (79.2%) in the absence of EGF, it was largely dimeric (87.1%) in the presence of EGF (Figure 2E and Tables 1 and 3). In contrast, although as expected, Tyr^251^Ala,Arg^285^Ser EGFR was indeed largely monomeric (94.2%) in the absence of EGF, this distribution was unaltered (monomer 91.7%) following the addition of the ligand (Figure 2F and Tables 1 and 3). These results are entirely consistent with the biochemical studies and demonstrate that both ligand-induced receptor dimerization and the lack of capacity of a mutationally modified transmembrane receptor to reach the dimeric configuration can be observed and quantified directly by SpIDA performed on simple confocal images.

**Figure 3.**
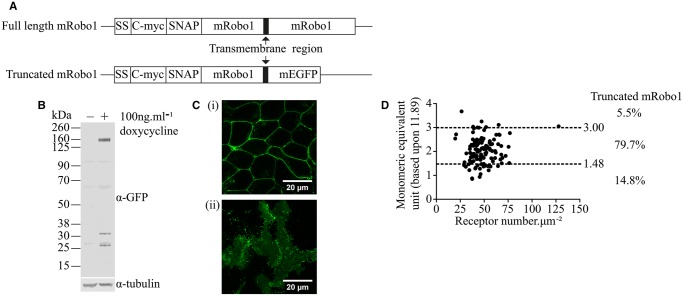
A Robo-1 receptor construct is predominantly dimeric. Cartoon illustrating the C-terminally truncated form of Robo-1 used in these studies and its relationship to the full-length protein (**A**). (**B**) Immunodetection of this construct following induction (+doxycycline) in Flp-In™ T-REx™ 293 cells. (**C**) Cross-sectional (**i**) and basolateral (**ii**) confocal images of these cells following induction of expression of the Robo-1 construct. Scale bar = 20 µm. (**D**) SpIDA analysis of the organizational state of the Robo-1 construct as in Figure 1. Proportions scored as monomer, dimer and oligomer are shown.

To extend these studies, we considered the axonal guidance receptor Robo-1. We have previously argued, based largely on analysis of FRET studies that, in the basal state, the full-length version of this receptor is largely dimeric [21]. In these previous studies, we also examined a C-terminally truncated version of Robo-1 (Figure 3A) which, although remaining largely dimeric, also displayed a monomeric fraction. When expressed, the full-length form generated a basolateral expression pattern that was difficult to assess via SpIDA (not shown); we, herein, therefore used the C-terminally truncated form, which did not present this issue. When induced from the Flp-In™ T-REx™ locus of Flp-In™ T-REx™ 293 cells harbouring a C-terminally mEGFP-tagged form of this construct (Figure 3A), it was expressed predominantly as an apparently 165 kDa polypeptide (Figure 3B) located at the plasma (Figure 3C(i)) and basolateral membranes (Figure 3C(ii)). QB analysis indicated this construct to display mean MEU = 2.04 ± 0.05, *n* = 128, with particle number in the range of 20–128 µm^−2^ (mean 48 ± 1.2) (Table 1). SpIDA analysis indicated that this receptor construct to be present predominantly as dimers (79.7%), with smaller proportions of monomers (14.8%) and potential oligomers (5.5%) (Figure 3D).

**Figure 4.**
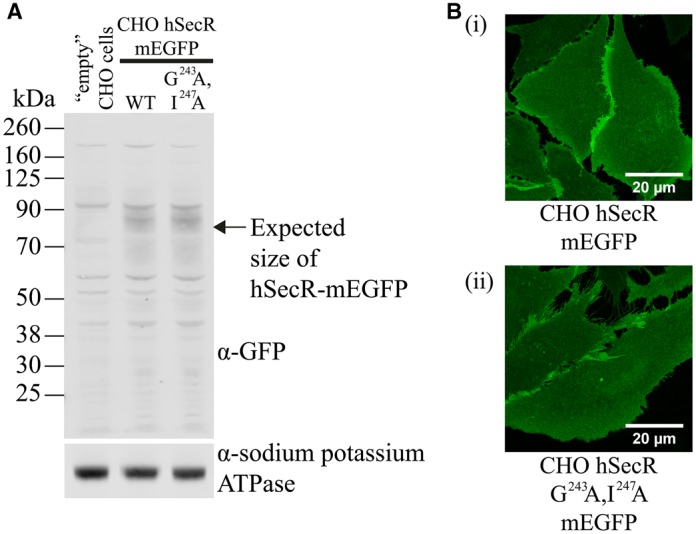
Expression of wild-type secretin receptor and Gly^243^Ala,Ile^247^Ala secretin receptor in CHO-K1 cells. Immunoblotting cell membrane preparations of non-transfected CKO-K1 cells or those expressing either wild-type secretin receptor-mEGFP or Gly^243^Ala,Ile^247^Ala secretin receptor-mEGFP with an anti-GFP antiserum (**A**, upper panel). Equivalent detection of Na^+^/K^+^ ATPase provided loading controls (**A**, lower panel). (**B**) Representative confocal images of the basolateral membrane of the receptor-expressing cells (**i**) wild-type secretin receptor and (**ii**) Gly^243^Ala,Ile^247^Ala secretin receptor. Scale bar = 20 µm.

**Figure 5.**
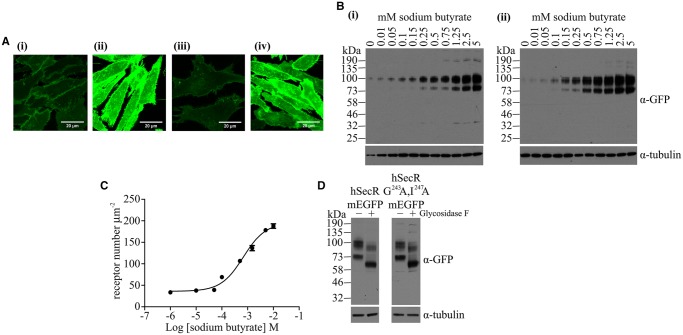
Treatment with sodium butyrate up-regulates levels of both wild-type and Gly^243^Ala,Ile^247^Ala secretin receptor. Images of the basolateral surface of CHO cells expressing the wild-type (**Ai,ii**) or Gly^243^Ala,Ile^247^Ala (**Aiii,iv**) secretin receptor that were untreated (**Ai,iii**) or treated overnight with 5 mM sodium butyrate (**Aii,iv**) are shown. (**B**) Samples of cell lysates generated from CHO cells expressing the wild-type (**i**) or Gly^243^Ala,Ile^247^Ala (**ii**) secretin receptor treated with the indicated concentrations of sodium butyrate were resolved by SDS–PAGE and immunoblotted with an anti-GFP antiserum. Corresponding immunodetection of tubulin provided loading controls. (**C**) Receptor levels assessed via quantal brightness analyses are shown following treatment with varying concentrations of sodium butyrate. (**D**) Samples as in (**B**) (treated with 5 mM sodium butyrate) were treated with or without *N*-glycosidase, resolved by SDS–PAGE and immunoblotted with an anti-GFP antiserum (**upper panels**). Corresponding immunodetection of tubulin (**lower panels**) provided loading controls.

Harikumar et al. [13] have previously used combinations of BRET and Bi-FC to argue that the secretin receptor, a class B GPCR, forms dimers but not higher-order oligomers. Furthermore, they generated evidence that residues within transmembrane domain IV, specifically Gly^243^ and Ile^247^, provide key determinants for this interaction [14]. Following in-frame linkage of mEGFP to the C-terminal tail of either the wild-type secretin receptor or a Gly^243^Ala,Ile^247^Ala mutant, CHO-K1 cell lines stably expressing each form were generated (Figure 4A). Images of the basolateral surface of these lines suggested similar distribution of the wild-type and mutant receptor (Figure 4B(i,ii)), and direct measures of mEGFP fluorescence indicated each form of the secretin receptor to be expressed as similar levels per cell (wild type = 1.09 ± 0.04 fluorescence units; Gly^243^Ala,Ile^247^Ala mutant = 1.00 ± 0.03 fluorescence units). Studies on many class A GPCRs, using either SpIDA [20] or single-molecule analysis [26], have indicated that oligomeric organization may increase with receptor density. This issue has not been assessed for any class B GPCR. Therefore, to consider this for both wild-type and the Gly^243^Ala,Ile^247^Ala secretin receptor mutant, and because these cells lacked a means to regulate receptor expression in an antibiotic-dependent manner, we treated cells expressing either form of the secretin receptor with sodium butyrate, which can activate viral promoters and enhance protein expression in stably transfected cells, when a viral promoter is used to express the protein, and has been shown in many cases to produce up-regulation of a GPCR of interest [27,28]. Overnight treatment of cells with 5 mM sodium butyrate resulted in extensive up-regulation of each form of the receptor, as shown using imaging studies that focused on the basolateral surface of the cells (Figure 5A). This effect was concentration-dependent over a broad range of sodium butyrate concentrations (10 µM to 5 mM) when assessed in immunoblotting studies using an anti-GFP antiserum (Figure 5B) or by employing QB and SpIDA to quantify receptor levels µm^−2^ (Figure 5C). Both the wild-type secretin receptor-mEGFP and the Gly^243^Ala,Ile^247^Ala secretin receptor- mEGFP mutant were detected as two resolvable species in SDS–PAGE (Figure 5B,D). These reflected differentially N-glycosylated species, as treatment with *N*-glycosidase F increased mobility of both forms and favoured production of the more rapidly migrating of these species (Figure 5D).

**Figure 6.**
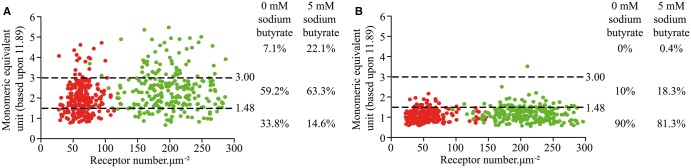
SpIDA analysis indicates the wild type secretin receptor to be a mixture of monomers and dimers whilst Gly^243^ Ala, Ile^247^ Ala secretin receptor is largely monomeric QB and SpIDA were performed on CHO-K1 cells expressing either the wild-type secretin receptor (A) or the Gly^243^Ala,Ile^247^Ala secretin receptor (B) that were either untreated (red) or treated overnight with 5 mM sodium butyrate (green). Proportions of data corresponding to monomer, dimer or oligomer are shown.

In the cells expressing wild-type secretin receptor but not treated with sodium butyrate, QB and SpIDA analysis on RoIs selected from confocal images indicated that, at the basolateral surface, the receptor was expressed at modest levels (63.6 ± 1.1 receptors µm^−2^, *n* = 240, mean ± SE), while equivalent analysis indicated Gly^243^Ala,Ile^247^Ala secretin receptor-mEGFP to be present at 56.3 ± 1.1 receptors µm^−2^, *n* = 240, mean ± SE (Table 2 and Figure 6). Although expressed at similar levels, when calculated and expressed as MEU, data derived from QB analysis indicated that dimeric/oligomeric organization of wild-type secretin receptor-mEGFP [MEU = 1.92 ± 0.05 (*n* = 240) (Table 2 and Figure 6A] was markedly greater than for Gly^243^Ala,Ile^247^Ala secretin receptor-mEGFP (MEU = 1.07 ± 0.02; *n* = 240; Table 2 and Figure 6B). SpIDA performed on these data sets indicated the wild-type receptor to be predominantly dimeric (59.2%) but a substantial fraction was identified as being monomeric (33.8%), while a small proportion (7.1%) was scored as oligomeric (Figure 6A). In contrast, the Gly^243^Ala,Ile^247^Ala mutant secretin receptor was assessed to be predominantly monomeric (90.0%), with the remaining 10.0% scored as dimeric (Figure 6B). Treatment with 5 mM sodium butyrate increased levels of the wild-type secretin receptor by some 3.15-fold (to 200.5 ± 2.7 receptors µm^−2^, *n* = 240, mean ± SE; Table 2). It also increased receptor organizational complexity (MEU = 2.42 ± 0.06, mean ± SE; Table 2), and this corresponded to a diminution in the proportion of monomeric receptor species (to 14.6%) and an increase in the proportion of oligomeric forms to 22.1% (Figure 6A). In contrast, although treatment with 5 mM sodium butyrate produced an even greater extent of up-regulation of Gly^243^Ala,Ile^247^Ala secretin receptor-mEGFP (3.69-fold; Table 2), this produced only a small increase in calculated population MEU (1.19 ± 0.02) (Table 2), consistent with the bulk of this variant remaining monomeric. A modest increase in the proportion of assessed dimers was also observed at the elevated expression level (Figure 6B), but only a single isolated observation was consistent with an oligomeric arrangement of the mutant receptor (Figure 6B).

**Table 2:** Details of analysis of mEGFP-tagged secretin constructs and treatments used

Construct and treatment	Number of RoIs analyzed	Mean MEU value	Range of expression (µm^−2^)	Average expression (µm^−2^)
hSecR-mEGFP (UT)	240	1.92 ± 0.05*	28–124	63.6 ± 1.1
hSecR-mEGFP (5 mM sodium butyrate)	240	2.42 ± 0.06^†^	76–287	200.5 ± 2.7
hSecR G^243^A I^247^A-mEGFP (UT)	240	1.07 ± 0.02	22–146	56.3 ± 1.5
hSecR G^243^A I^247^A-mEGFP (5 mM sodium butyrate)	240	1.19 ± 0.02^‡^	95–296	207.9 ± 2.5
hSecR-mEGFP (UT)	240	1.92 ± 0.04	29–206	83.5 ± 2.6
hSecR-mEGFP (5 µg ml^−1^ cholera toxin)	240	1.85 ± 0.05	16–191	75.1 ± 3.1
hSecR-mEGFP (UT)	210	2.08 ± 0.06	23–237	79.1 ± 2.3
hSecR-mEGFP (100 ng ml^−1^ secretin)	210	2.23 ± 0.06	29–159	80.0 ± 1.8
hSecR G^243^A I^247^A-mEGFP (UT)	210	1.25 ± 0.03	27–200	81.8 ± 2.3
hSecR G^243^A I^247^A-mEGFP (100 ng ml^−1^ secretin)	210	1.43 ± 0.04	9–240	72.5 ± 2.3

Abbreviation: UT, untreated.

* Greater than hSecR G^243^A, I^247^A-mEGFP (*P* < 0.0001).

† Greater than hSecR-mEGFP UT (*P* = 0.0005).

‡ Greater than hSecR G^243^A, I^247^A-mEGFP UT (*P* = 0.004).

**Figure 7.**
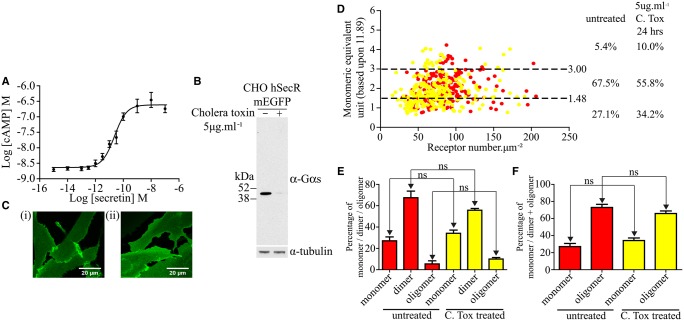
Substantial down-regulation of Gα_s_ does not have an impact on secretin receptor quaternary organization. CHO-K1 cells expressing the wild-type secretin receptor were treated with various concentrations of secretin and cAMP levels measured. A representative example of *n* = 5 is shown (**A**). (**B**) CHO-K1 cells expressing the wild-type secretin receptor were then treated with vehicle or with cholera toxin for 24 h. Lysates from these cells were then immunoblotted to detect Gα_s_ (**upper panel**). Immunoblotting for tubulin provided loading controls (**lower panel**). (**C**) Confocal images of the basolateral surface of cells treated with vehicle (**i**) or cholera toxin (**ii**) are shown. Scale bar = 20 µm. (**D**) QB analysis and SpIDA were performed on such samples. Each data point represents a unique RoI. Untreated, red; cholera toxin-treated, yellow. Proportions of predicted monomer, dimer and oligomer are shown. (**E** and **F**) Statistical analysis of the outcomes: (**E**) calculated based on % of monomer, dimer and oligomer and (**F**) calculated based on % of monomer versus dimer + oligomer. ns, not significantly different.

The secretin receptor couples predominantly to the G-protein Gαs to cause elevation of cAMP levels upon the addition of the hormone secretin, and in these cells did so with pEC_50_ 10.63 ± 0.10 (mean ± SEM, *n* = 5) for the hormone (Figure 7A). As Gαs is clearly able to interact with the secretin receptor as part of a signalling complex, we then considered whether the G-protein might contribute to stability of the dimeric form of the receptor. Although cholera toxin is most often considered as an activator of Gαs and therefore of adenylyl cyclase activity and cAMP levels, sustained treatment of cells with this toxin is well known to cause a marked down-regulation of Gα_s_ levels [29,30]. After treating cells expressing the wild-type secretin receptor with cholera toxin (5 µg ml^−1^, 24 h), extensive down-regulation of Gα_s_ was confirmed in immunoblotting studies (Figure 7B). This did not intrinsically appear to alter the basolateral distribution of the receptor (Figure 7C). QB analysis and SpIDA confirmed that treatment with cholera toxin did not affect levels of the receptor (untreated 83.5 ± 2.6 molecules µm^−2^, treated 75.1 ± 3.1 molecules µm^−2^) (Table 2) or its distribution profile (Figure 7D). Moreover, although treatment with cholera toxin did appear to result in small shifts in the dimer/monomer proportions of the secretin receptor to favour the monomeric state, this did not reach statistical significance (Figure 7E,F). These studies indicate that although it would certainly have been a reasonable prediction that availability of cognate G-protein could contribute to dimer stability, in the case of the secretin receptor this plays a minimal role and again suggests that key interaction affinity is provided by residues of transmembrane domain IV, with particular roles for Gly^243^ and Ile^247^.

**Figure 8.**
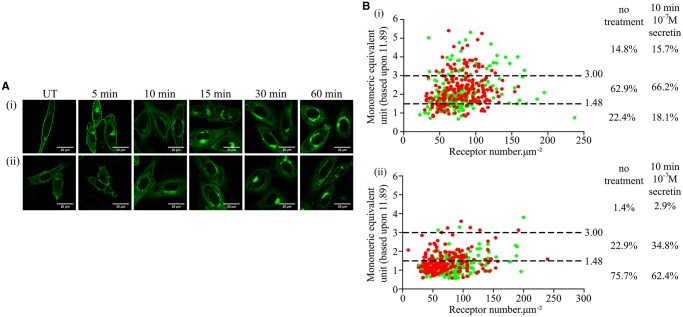
Short-term treatment with secretin does not alter the quaternary organization of the wild-type secretin receptor. CHO-K1 cells expressing the wild-type (**Ai**) or Gly^243^Ala,Ile^247^Ala (**Aii**) secretin receptors untreated (UT) or treated with 100 nM secretin for the indicated times. They were then fixed and imaged to identify receptor attached mEGFP. (**B**) Wild-type (**Bi**) or Gly^243^Ala,Ile^247^Ala (**Bii**) cells were treated for 10 min with vehicle (**green**) or 100 nM secretin (**red**); images were analyzed by SpIDA; each data point represents a unique RoI. Proportions of predicted monomer, dimer and oligomer are shown.

Finally, we assessed if treatment with secretin might alter the organizational structure of the secretin receptor-mEGFP constructs. A concentration of secretin (100 nM) substantially greater than needed to fully activate cAMP production (Figure 7A) was added to cells expressing either wild-type secretin receptor-mEGFP or Gly^243^Ala,Ile^247^Ala secretin receptor-mEGFP, and the cellular distribution of the mEGFP tag was monitored over time. Substantial levels of internalization of both forms of the receptor were observed at time points beyond 15 min (Figure 8A). To assess if secretin produced rapid effects on receptor organizational structure, we therefore fixed cells and employed SpIDA 10 min after the addition of secretin (Figure 8B). No statistically significant effect of the ligand was observed on the proportions of monomer, dimer or oligomer forms for the wild-type receptor (Figure 8B(i)), while a statistically significant increase in mean MEU (*P* = 0.0004) (Table 2) was detected for Gly^243^Ala,Ile^247^Ala secretin receptor-mEGFP, corresponding to an increase in dimeric, and corresponding reduction in monomeric, forms (Figure 8B(ii)) of the mutated receptor.

## Discussion

The tendency for GPCRs to associate with themselves or with other family members is broadly aligned with receptor family, with the class C, glutamate-like GPCRs forming the most stable, essentially obligate dimers/oligomers [3], the class A GPCRs often associating quite transiently [7] and via diverse interfaces, and the class B GPCRs believed to be intermediate between the other two classes [1,2]. The prototypic class B secretin receptor has been reported to associate along the lipid face of transmembrane domain IV to form homodimers, with no contribution of either extracellular or intracellular regions of this receptor [13,14]. These dimeric complexes can be destabilized or disrupted by competition with a peptide corresponding to the sequence of transmembrane domain IV or by mutating at least two residues along the lipid face of this helix in an intact secretin receptor construct [14]. Thus, while it is known that the secretin receptor is capable of forming and existing as homodimeric complexes, the percentage of receptors involved in such complexes relative to those in monomeric form has not been established, and the techniques used to date to examine the quaternary organization of the secretin receptor are poorly suited to assess this.

In the currently reported studies, we clearly demonstrate the power of SpIDA [18,19] to determine the percentage of a population of cell surface receptors at steady state that remain free as monomers versus those present as dimeric complexes. As proof of concept, this was very clearly validated using the EGFR, known to form dimeric complexes in an agonist-dependent manner that are required for biological activity. The ability to disrupt agonist-induced formation of such complexes using a well-established EGFR mutant [23] was similarly demonstrated.

In applying the SpIDA methodology to a class B GPCR receptor for the first time, we demonstrate that under stable, equilibrium conditions and with modest expression levels, some 60% of the secretin receptors on the cell surface are involved in homodimeric complexes, with the remainder predominantly in a monomeric form. This is substantially higher than has been reported for many class A GPCRs [7,22,26], but lower than for the class C GPCRs where dimerization/oligomerization appears to be an intrinsic and integral feature of their structure and mode of action [3]. Few, if any higher-order secretin receptor complexes were identified using this technique at lower expression levels and this may reflect that the interface appears to be formed specifically by symmetrical transmembrane domain IV–transmembrane domain IV interactions [14] and that this organizational structure may well be common amongst class B GPCRs [11,31,32]. While larger oligomeric complexes of many class A receptors have been observed [1,2], this appears to be highly receptor concentration-dependent [20,26] and that, for different members of the family, distinct transmembrane domains, and even regions out-with the core seven-transmembrane domain structure of the receptors, may contribute to such interactions [1,2]. Like the class A GPCRs, induction of high levels of expression of the secretin receptor by the treatment of cells with a maximally effective concentration of sodium butyrate did result in the appearance of a significant proportion of oligomeric species, but the molecular basis for this organization remains to be explored. It was also shown that mutating residues along the lipid face of transmembrane domain IV of the secretin receptor was capable of disrupting the dimeric complexes, resulting in an almost exclusive presence of monomeric forms of this receptor. This, too, provides support for a single, structurally well-defined interface determining the production of secretin receptor homodimers, with the ability to disrupt such complexes by engineering changes in that single region [14]. Little is currently understood about the dynamics of complex formation for the class B GPCRs. The current demonstration of an equilibrium between secretin receptor monomers, homodimers and oligomeric forms suggests that there is probably a stable rate of association and dissociation of these complexes. However, it remains unclear what these rates are or what might influence them.

An unanswered issue in many studies of GPCR organizational state, despite a large number of reports on the topic [1], is the effect of ligands on the distribution between these forms. Recently, for both the serotonin 5-HT2c receptor [20] and the muscarinic M_1_ acetylcholine receptor [22], we have used SpIDA to show that certain, but not all chemical classes, of antagonists at these receptors can either increase [22] or decrease [20] the proportion of receptors within such complexes. There are no useful small-molecule antagonists of the secretin receptor. However, although sustained treatment with the agonist ligand secretin causes internalization of both the wild-type and Gly^243^Ala,Ile^247^Ala mutant secretin receptor, and this can confound analysis and interpretation of SpIDA, we show that, at least prior to receptor internalization, interaction with secretin does not alter the structural organization of the wild-type receptor. Interestingly, we did note a significant increase in the proportion of the Gly^243^Ala,Ile^247^Ala mutant detected as being dimeric. Such data must be interpreted with caution, however. Although it is clear from studies with the Gly^243^Ala,Ile^247^Ala mutant that the secretin receptor can internalize from the cell surface as a monomer, the observed increase in ‘dimeric’ species may reflect clathrin-induced receptor proximity rather than genuine-enhanced protein–protein interactions between the receptor monomers.

It is important to appreciate that unlike methods based on single-molecule detection [7,26,33–36], SpIDA provides a statistical sampling of fluorescent properties of suitably labelled molecules within individual RoIs [18] and, in so doing, indicates the likelihood of monomers, dimers or oligomers predominating in each RoI. As such there is a need to sample many RoIs and to apply statistical inference to reach conclusions. As noted in Results, analysis of the distribution of measured QB values for both PM-1-mEGFP and PM-2-mEGFP showed these to be normally distributed. As such, definition of ‘larger than monomer’ in the experimental studies, as those observations having QB > 1.48 MEU and >3.00 MEU as being ‘oligomeric’ reflect selection above 1.5 SD greater than the mean QB for the fluorescent molecule used for calibration. This gate could have been set at an even more stringent level, e.g. 2.0. SD above the mean value, i.e. >1.64 MEU, to be scored as dimeric. This would have altered absolute estimates of percentages of monomer versus dimer, i.e. for the EGFR, the same data set would have returned 85% monomer and 15% dimer in the basal state and only 78.3% dimer and 20.8% monomer in the presence of the agonist ligand (Table 3); this would not change the conclusion that binding of EGF moves the monomer–dimer equilibrium substantially towards the dimer. Similarly, although providing slightly different percentages of monomer to dimer for the secretin receptor, this would have not altered and, indeed, would have further strengthened, the conclusion that the Gly^243^Ala,Ile^247^Ala is largely monomeric.

**Table 3:** Analysis of monomer to dimer proportions for the unoccupied and EGF-stimulated EGFR estimated at different statistical stringency thresholds.

Cell line	Treatment	Quaternary structure	1.5× standard deviations	2× standard deviations
EGFR-mEGFP	UT	Monomers	79.2% [<1.48 MEU]	85% [<1.64 MEU]
		Dimers	20.8% [>1.48 MEU]	15% [>1.64 MEU]
		Oligomers	0% [>3.00 MEU]	0% [>3.33 MEU]
EGFR-mEGFP	100 nM EGF 15 min	Monomers	10.8%	20.8%
		Dimers	87.1%	78.3%
		Oligomers	2.1%	0.8%
EGFR Y^251^A,R^285^S-mEGFP	UT	Monomers	94.2%	96.7%
		Dimers	5.4%	2.9%
		Oligomers	0.4%	0.4%
EGFR Y^251^A,R^285^S-mEGFP	100 nM EGF 15 min	Monomers	91.7%	96.7%
		Dimers	8.3%	3.3%
		Oligomers	0%	0%

Abbreviation: UT, untreated.

Possible contributors to the stability of GPCR complexes are GPCR-interacting proteins, with the most obvious being the predominant G-protein that interacts with the receptor to allow signal transduction to occur. In the case of the secretin receptor, this is the cAMP-stimulatory G-protein G_s_. By treating cells with cholera toxin for a sustained period, Gα_s_ is down-regulated [29–30]. Here, immunoblotting to detect Gα_s_ showed this process to be very effective. Despite this SpIDA was able to demonstrate that this manoeuvre had no substantial impact on the state of association of the secretin receptor. Importantly, while the homodimeric secretin receptor complex has been reported to be functionally important, contributing to high-affinity binding and agonist biological activity [11,14], this suggests that the G-protein association event is not required for formation or stabilization of this complex.

Observations of internalization of the non-ligand-occupied GPCR along with the ligand-occupied GPCR involved in class B GPCR heterodimers suggest a relatively prolonged presence of such structures [37]. In another report, co-expression of GIP receptors that did not internalize in response to GIP with the GLP-1 receptors that did internalize in response to their natural ligand was able to slow or inhibit the latter process [38]. This also supports the prolonged presence of these complexes, but will need to be studied more extensively for other class B family members. As with other GPCR classes, agonist-induced internalization of class B receptors, including the secretin receptor, appears to occur via clathrin-mediated endocytosis and involves a key role for arrestin isoforms that interact with the receptor in an agonist-dependent manner. As presently implemented, SpIDA effectively samples and assesses the organization of fluorescent complexes at the basolateral surface of cells in contact with a glass coverslip; then proteins that move away from this surface and internalize within the cell become invisible to the analysis. As such, although we were able to demonstrate that relatively short-term addition of a receptor-saturating concentration of secretin did not alter the organizational structure of the wild-type receptor, it would be of great interest to assess if the secretin receptor remains predominantly dimeric when internalization of the agonist-occupied receptor is achieved.

## Abbreviations

Bi-FC: bi-molecular fluorescence complementation
BRET: bioluminescence resonance energy transfer
cAMP: cyclic AMP
CHO: Chinese hamster ovary
EGFP: enhanced green fluorescent protein
EGFR: epidermal growth factor receptor
FRET: fluorescence resonance energy transfer
GPCR: G-protein-coupled receptor
HBSS: Hanks balanced salt solution
mEGFP: monomeric enhanced green fluorescent protein
MEU: monomeric equivalent unit
MOPS: 3-Morpholinopropane-1-sulfonic acid
PBS: phosphate-buffered saline
QB: quantal brightness
RET: resonance energy transfer
RoI: region(s) of interest
SpIDA: spatial intensity distribution analysis
